# EV-A71 invades the central nervous system and affects the blood-brain barrier in a tree shrew model

**DOI:** 10.3389/fimmu.2025.1583768

**Published:** 2025-06-26

**Authors:** Wen-Guang Wang, Xuan Wang, Na Li, De-Xuan Kuang, Pin-Fen Tong, Cai-Xia Lu, Yuan-Yuan Han, Xiao-Mei Sun, Jie-Jie Dai, Long-Ding Liu

**Affiliations:** Institute of Medical Biology, Chinese Academy of Medical Sciences and Peking Union Medical College, Kunming, Yunnan, China

**Keywords:** enterovirus A71, tree shrew, infection, neurotropism, blood brain barrier

## Abstract

**Introduction:**

The enterovirus A71 (EV-A71)-caused central nerve system (CNS) damage seriously endangers the health of infants and young children, but the underlying mechanisms by which EV-A71 crosses the blood-brain barrier (BBB) are still largely unknown. This study developed a tree shrew (*Tupaia belangeri*) model to examine EV-A71 neurotropism and the disruption of the BBB.

**Methods:**

A cohort of twenty-two tree shrews, aged three months, were inoculated intranasally and orally with EV-A71 to establish an *in vivo* infection model. Complementary *in vitro* experiments were conducted using primary brain microvascular endothelial cells (MVECs) and astrocytes (AS) isolated from tree shrews.

**Results:**

Tree shrews infected with EV-A71 demonstrated symptoms of fever, vesicular lesions, and sustained viremia. Viral replication was observed in neural tissues, including the brain and spinal cord, as well as in select non-neural organs, accompanied by histopathological changes. Evans blue permeation assessment showed increased BBB permeability. EV-A71 infection down-regulated tight junction proteins Claudin-5 and junctional adhesion molecule A in the brain. *In vitro* studies showed that EV-A71 replicated efficiently in MVECs and AS, inducing cytopathic effects. Scavenger Receptor Class B Member 2 (SCARB2) and Annexin A2 (ANXA2) were identified as potential functional receptors facilitating viral entry. EV-A71 infection led to the dysregulation of tight junction proteins, matrix metalloproteinases, and Major facilitator superfamily domain-containing protein 2a. EV-A71 also stimulated the immune activity of AS.

**Discussion:**

This study indicated that SCARB2 and ANXA2 play a role in the invasion of EV-A71 into the CNS of tree shrews. EV-A71 infection down-regulated tight junction proteins and increased the BBB permeability. This model provides a novel platform for studying EV-A71 neuropathogenesis.

## 1 Introduction

Enterovirus A71 (EV-A71), a member of the enterovirus genus of the *Picornaviridae* family, is one of the main pathogens that causes hand, foot and mouth disease (HFMD) in infants and young children. In severe cases, EV-A71 also causes aseptic meningitis, brainstem encephalitis, acute flaccid paralysis (AFP) and some other neurological complications. EV-A71 has broken out in different regions of the world (1–3), and it has become more frequent in the Asia-Pacific region in recent years (4–6).

Numerous studies have established that EV-A71 has the capability to induce central nervous system (CNS) damage (7–10), yet the precise mechanism by which the virus gains entry into the CNS remains uncertain. Neurotropic viruses, including human immunodeficiency virus (HIV) and poliovirus (PV), have been shown to utilize retrograde axonal transport or direct penetration of the blood-brain barrier via the bloodstream to infiltrate the CNS (11). Evidence of retrograde axonal transport in mouse models of EV-A71 infection has been documented (12). However, EV-A71 demonstrated pronounced muscle tropism after adaptation in mice, which is rarely observed in fatal human cases (13). Scavenger receptor class B member 2 (SCARB2) is essential for EV-A71 infection, but mouse SCARB2 is ineffective in adult mice (14). While neonatal mice exhibit susceptibility to EV-A71, robust and reproducible mouse infection models necessitate transgenic expression of human SCARB2 (15). Consequently, it is plausible that the mechanisms elucidated in mouse models may diverge from those observed in human hosts.

While there is limited documentation of EV-A71 directly crossing the blood-brain barrier, viremia has been consistently observed in various infection models, such as mice (16, 17), gerbils (18), tree shrews (19), and monkeys (20). *In vitro* experiments have also confirmed that EV-A71 can directly infect brain microvascular endothelial cells, astrocytes and neuronal cells (7, 8). Therefore, it will make sense to explore the possibility of EV-A71 invading CNS through the blood-brain barrier.

The tree shrew is a new type of laboratory animal that has a close relationship to nonhuman primates (21), and it has been widely used in the establishment of human disease models (22), particularly for viral infections such as HSV-1 (23), Zika (24) and even SARS-CoV-2 (25–27). Our previous studies have found that EV-A71 can infect tree shrews, and symptoms similar to HFMD and AFP were observed, suggesting that tree shrew may serve as a valuable model for investigating the CNS tropism of EV-A71.

The objective of this study was to explore whether EV-A71 infection affects the CNS of tree shrews, which might include how EV-A71 enters the CNS and affect the BBB. In this work, tree shrews and the brain microvascular endothelial cells and astrocytes were used to investigate the neurotropism of EV-A71. The results showed that EV-A71 can infect the CNS of tree shrews and increase the permeability of the BBB.

## 2 Materials and methods

### 2.1 Animals and ethics statement

Tree shrews (*Tupaia belangeri chinensis*) were bred in the Center of Tree Shrew Germplasm Resource (Use license: SYXK K2018-0002; Manufacturing license: SCXKK2018-0002), the Institute of Medical Biology, Chinese Academy of Medical Sciences. All animal procedures were approved by the Institutional Animal Care and Use Committee of the Institute of Medical Biology, Chinese Academy of Medical Science (Ethics number: DWSP201801 002). The infection experiments were carried out in Animal Biosafety Level 2 facilities (20–24°C temperature; 40–60% humidity; −30 ± 10 pa negative air pressure; 12 h:12 h light: dark cycle). All animal experiments were carried out in accordance with the ARRIVE guidelines.

### 2.2 Cells

Vero cells (African green monkey kidney cells), tree shrew brain microvascular endothelial cells (MVECs) and astrocytes (AS) were preserved by the Tree Shrew Germplasm Resource Center, Institute of Medical Biology, Chinese Academy of Medical Sciences. Human brain microvascular endothelial cells (HBMECs) were purchased from BeNa Culture Collection (Beijing, China).

The medium for Vero cells was DMEM (HyClone, USA) +10% FBS (Gibco, USA) + penicillin (100 IU/mL) and streptomycin (100 μg/mL) (HyClone, USA). The medium for AS was DMEM/F12 (HyClone, USA) +10% FBS (Gibco, USA) + penicillin (100 IU/mL) and streptomycin (100 μg/mL) (HyClone, USA). For MVECs and HBMECs, 1% ECGS (ScienCell, USA) was added to the AS medium. Cells were incubated at 37 °C, 5% CO_2_.

### 2.3 Virus

EV-A71 (FY-23, subgenotype C4, GenBank: EU812515.1) was isolated and gifted by the Viral Immunology Laboratory of the Institute of Medical Biology, Chinese Academy of Medical Sciences.

Vero cells were used for virus infection and titer determination. Cells grown to approximately 80%–90% confluence were infected with EV-A71 at a multiplicity of infection (MOI) of 0.1 or 0.5. After 1 h of adsorption, the virus was washed off with PBS, and the cells were maintained in serum-free medium for 24 h or 48 h.

The virus used in animal infection experiments was cultured in Vero cells, and the titer was determined to be 8×10^6^ TCID_50_/mL by the Reed-Muench method.

All virus cultivation experiments were carried out in a biosafety level 2 laboratory.

### 2.4 Antibodies

EV-71-VP1 polyclonal antibody (GeneTex, GTX132339, USA), beta actin antibody (GeneTex, GTX109639, USA), Annexin A2 (ANXA2) antibody (GeneTex, GTX101902, USA), SCARB2 (LIMP II) antibody (GeneTex, GTX53371, USA), Junctional adhesion molecule A (JAM-A) antibody (GeneTex, GTX01112, USA), Claudin-5(CLDN5) antibody (GeneTex, GTX00796, USA), Occludin (OCLN) antibody (GeneTex, GTX85016, USA)), Claudin 1(CLDN1) antibody (GeneTex, GTX134842, USA), Matrix Metalloproteinase 9(MMP-9) antibody (GeneTex, GTX100458, USA), Glyceraldehyde-3-Phosphate Dehydrogenase (GAPDH) antibody (GeneTex, GTX GTX100118, USA), Alexa Fluor 488-conjugated goat anti-mouse IgG (Abcam, ab150113, USA), Alexa Fluor 647-conjugated goat anti-rabbit IgG (Abcam, ab150083, USA), goat anti-rabbit IgG antibody (HRP) (GeneTex, GTX213110-01, USA) and goat anti-mouse IgG antibody (HRP) (GeneTex, GTX213111-01, USA).

### 2.5 Animal experiments and laboratory tests

**Figure 1:**
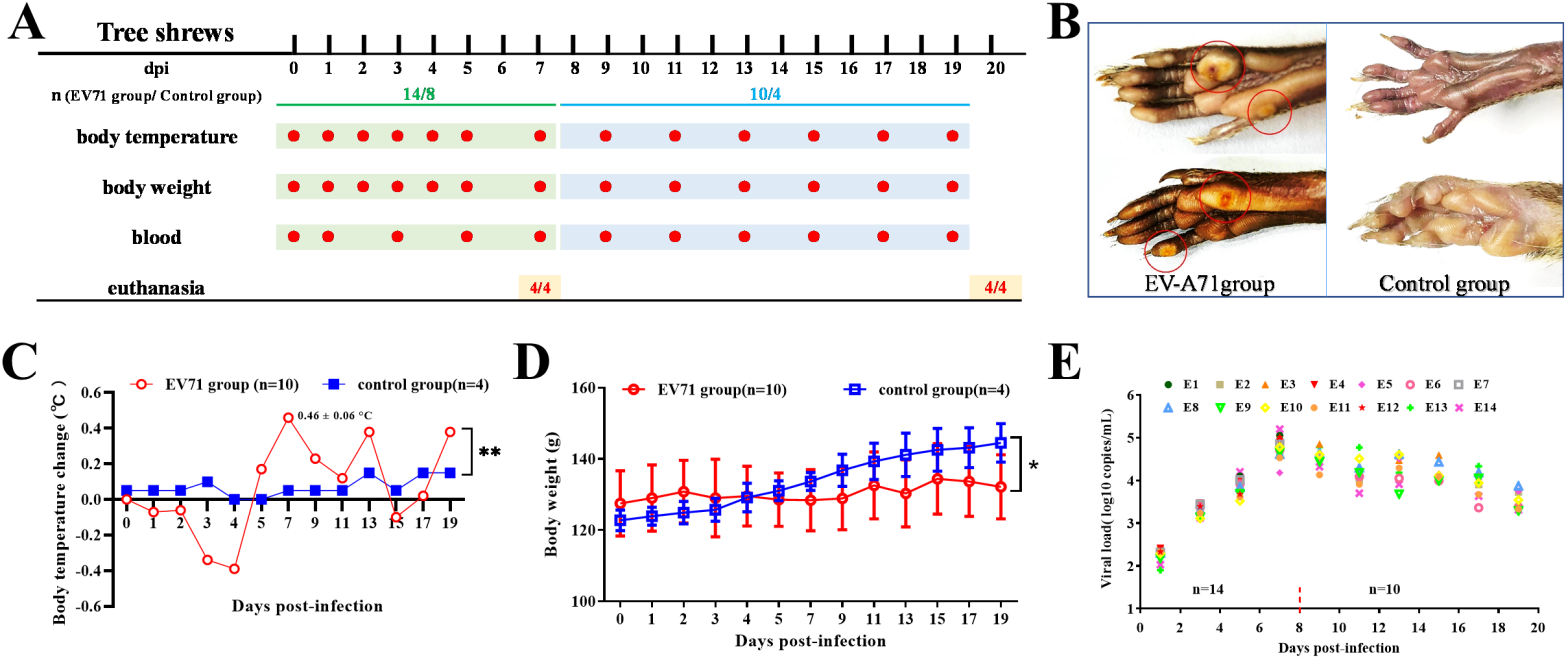
Study design, clinical signs and viral load in blood. **(A)** Study design: Three-month-old tree shrews were divided into two groups: the experimental group (n=14) and the control group (n=8). Each group included half male and half female. Each animal in the experimental group was given 1.2 mL (9.6×10^6^ TCID_50_) of EV-A71 by gavage (1 mL) and by nasal drip (200 μL). The control group was treated with cell culture medium. **(B)** Vesicular lesions in the feet. **(C)** Body temperature change (Two-way ANOVA, **P<0.01). **(D)** Body weight (Two-way ANOVA, *P<0.05). **(E)** Viral load in blood of the EV-A71-infected group.

The animal experimental procedures are outlined in **Figure 1A**. A total of 22 three-month-old (childhood) tree shrews were used in the *in vivo* infection experiment, half male and half female, weighing 120 ± 20 g. According to the random number table method, 8 tree shrews were selected as the normal control group, and the other 14 were the experimental group. Before infection, all basic data (body temperature and weight) and blood samples were collected. The titer of the EV-A71 virus was 8×10^6^ TCID_50_/mL. Each animal in the experimental group was given 1 mL (8×10^6^ TCID_50_) using a 16-gauge lavage needle and 200 μL (1.6×10^6^ TCID_50_) by nasal drip. The control group was subjected to mock infection using a sterile medium that did not contain the virus.

After infection, the body temperature (rectal temperature) and weight were measured once a day for the first 5 days and then every other day. Blood samples (500 μL) were collected from tail vein every other day, and centrifuged serum was used for viral load detection. At 7 days and 20 days post inoculation (dpi), 4 animals in each group were euthanized for viral load testing, western blot analysis, histopathological examination and Evans Blue (EB) dye extravasation experiments. The euthanasia procedure was intraperitoneal injection of overdose sodium pentobarbital 120 mg/Kg. At the end of the experiment, the rest of the animals were given biosafety disposal after euthanasia.

### 2.6 Quantification of viral load and the relative expression of target genes

RNAiso Blood (TaKaRa, 9113, Japan) was used to extract total RNA from serum, and RNAiso Plus (TaKaRa, 9109, Japan) was used to extract total RNA from tissues and cells. A One Step PrimeScript™ RT-PCR Kit (Perfect Real Time) (TaKaRa, RR064A, Japan) was used to detect the viral load. Quantified plasmids containing the target gene sequences of EV-A71 were used as standards. Standards were serially diluted from 10^9^ to 10^5^ copies/mL, and these were included in each TaqMan RT-qPCR run to generate standard curves (**Supplementary Figure 1**), with a detection limit of 25 copies. A One Step TB Green^®^ PrimeScript™ PLUS RT-PCR Kit (Perfect Real Time) (TaKaRa, RR096A, Japan) was used to detect the relative expression of target genes, using GAPDH as an internal control. RT-qPCR was performed using a Real Time PCR System (Bio-Rad, CFX-96, USA). The relative mRNA expression was measured by RT-qPCR using the 2 (-ΔΔCt) method (28).

The primers used in the above reaction are shown in **Table 1** and were designed and validated by the authors.

**Table 1:** Primers for RT-qPCR.

Primer name		Primer sequence
ZO-1	F	5’- AGACACAGACACTGAAGGCG -3’
	R	5’- CCTGGTTCTCGTGATGCACT -3’
ANXA2	F	5’- GTCTAGTCTCTCCTTTAAGC -3’
	R	5’- CCGTTTATTTATCTGTTTATGAC -3’
MMP-2	F	5’- CCAGACAGGTGACCTTGACC -3’
	R	5’- ACTTGGAAAGCTCGAGCGAA -3’
MMP-9	F	5’- CGGAGACGGGTATCCTTTCG -3’
	R	5’- GCCTAGCGTCCACAACTCTT -3’
GAPDH	F	5’-GTCAGCAATGCCTCCTGCAC-3’
	R	5’-GGGCCGTCCACAGTCTTCTG-3’
CLDN1	F	5’- TAGCCACAGAATAGTTCAAGA -3’
	R	5’- AAAGAGCCTGACCAAACT -3’
CLDN5	F	5’- AGGACCCAGCGTTTCCTTTT -3’
	R	5’- GGGAAGCAGCTACAGTCTGG -3’
OCLN	F	5’- CAACTGCTCAGGCTTCTGGA -3’
	R	5’- TACAATGGCAATGGCCTCCT -3’
MFSD2A	F	5’- GGATACGCCTTGTCTCCAGG -3’
	R	5’- TGCGTTTTGCGTGTCTTTGA -3’
CAV-1	F	5’-AAGGCATTCTGGGGACAAGG-3’
	R	5’-AGTGTAGAGATGTCCCCCCGC-3’
JAM-A	F	5’- CCCGTGAAGTCAGAAGCTGT -3’
	R	5’- GGGCTGGCTGTAAATCACCT -3’
SCARB2	F	5’- CCCTACATCATCATGGCGCT -3’
	R	5’- CAGGCGATGGTTAGGTTCGT -3’
IL-6	F	5’-GTGCCAGAGGTGTGCAGATT-3’
	R	5’-GACTGCATCTTCACCATCAAGC-3’
IL-8	F	5’-CGGAAGGAACCATCTCGCTC-3’
	R	5’-GGCAAAACTGCACCTTCACAC-3’
IFN-α	F	5’-GCTGAATGACCTGGAAGCCTGTG-3’
	R	5’-TCCTCACAGCCAGAATGGAGTCC-3’
IFN-β	F	5’-GCTCCAGCAGATCTTTGGCAT-3’
	R	5’-GCCCATCGAGTTCCACAAGG-3’
TNF-α	F	5’ -GGATGCCTACTGCTACAAC-3’
	R	5’ -ATACGCTGACCATACTTGAG-3’
EV-A71	F	5’ - CAGTCATCGATTGGATAC-3’
	R	5’ -CTCTGCTGAAGAAACTATC-3’
	Probe	5’ -FAM-CAAGGTTCCAGCACTCCAAGC-BHQ1-3’

### 2.7 Evans Blue extravasation test

The permeability of BBB was evaluated by fluorescence observation of frozen tissue sections and Evans Blue (EB) content determination of tissue (29, 30). EB dye solution (with a concentration of 2%, prepared with saline; the dosage was 2 mL/kg; Sigma, E2129, USA) was injected through the tail vein of the tree shrew, and the tree shrew was euthanized 1 h later. After whole-body perfusion with PBS, whole brain and spinal cord tissues were taken and immediately divided into several portions. One portion was quick-frozen with liquid nitrogen and sliced with a cryostat. The fluorescence intensity and range of EB were observed using a fluorescence microscope system (Nikon, ECLIPSE Ti, Japan). Five photographs were taken for each section, covering different areas from the edge to the center, and the average fluorescence intensity was calculated by ImageJ software (Ver 1.51). After being weighed, another portion of the tissue was incubated with formamide at 60°C for 24 h, then centrifuged at 6000 r/min for 5 minutes. The absorbance of the supernatant was determined at 620 nm with an enzyme-labeled instrument. The EB content in the sample tissue was calculated according to the standard EB spectrophotometry curve. BBB permeability was presented by the content of EB, which is equal to the concentration of EB over the brain wet weight (μg/g).

### 2.8 Histopathological and immunohistochemical examination

After whole-body perfusion with PBS, heart, liver, spleen, lung, kidney, muscle, sciatic nerve, brain, and spinal cord tissue samples were collected and fixed in 10% neutral formalin buffer. The paraffin sections were prepared using conventional methods. The slicing method for the brain and spinal cord was the same as the frozen sections in the previous step. Paraffin-embedded tissues were cut into 5 μm sections. After hematoxylin and eosin staining, the cell nucleus was stained blue, and the cytoplasm was red.

After deparaffinization and rehydration, citric acid (pH 9.0) antigen retrieval was performed. Then, the sections were placed in 3% hydrogen peroxide and incubated at room temperature (RT) in darkness for 25 minutes to block endogenous peroxidase activity. The sections were blocked with 3% Bovine Serum Albumin (BSA) for 30 minutes at RT. Sections were then treated overnight at 4°C with primary antibodies. After the sections were washed and dried, the tissues were covered with secondary antibody (HRP labeled) from the corresponding species of primary antibody and incubated at RT for 50 minutes. Immunohistochemical staining was performed with DAB and counterstained with hematoxylin. The EV-A71 antibody (GTX132338), ANXA2 (GTX101902) antibody, SCARB2 (LIMP II) antibody (GTX53371), JAM-A antibody (GTX01112), CLDN5 antibody (GTX00796), CLDN1 antibody (GTX134842) and OCLN antibody (GTX85016) were all diluted at 1:800. For negative controls, primary antibodies were replaced by normal rabbit IgG or normal mouse IgG. Slides were observed and photographed under a Nikon Eclipse Ci-L microscope. Sections were blindly analyzed by an independent pathologist. H-SCORE is a scoring method for tissue immunohistochemical results that reflects the positive ratio and the positive intensity (31, 32). H-SCORE was used to evaluate the expression of EV-A71 and other proteins.

### 2.9 Immunofluorescence observation

Cells were fixed with 4% paraformaldehyde for 20 min, and treated with 0.5% Triton X-100 at RT for 20 min, and then blocked with 5% goat serum at RT for 30 min. After washing with PBS, the prediluted primary antibody was added and incubated overnight at 4°C. The EV-A71 antibody (1:1000) was from rabbits, and the receptor antibody (1:800) was from mice. PBS was added to control wells instead of primary antibody. After washing with PBS, the corresponding fluorescent secondary antibody (1:1000) was added and incubated at 37°C for 1 h in the dark, and then DAPI was added and incubated for 2 min. Cell images were digitally captured using a fluorescence microscope system (Nikon, ECLIPSE Ti, Japan) (33).

Paraffin sections were dewaxed and hydrated with xylene and alcohol. The sections were placed in EDTA antigen retrieval buffer (pH = 8.0) for antigen repair, rinsed with PBS 3 times, 5 min each time, immersed in 3% H_2_O_2_ and incubate at room temperature for 15 min, rinsed with PBS 3 times, blocked with 5% BSA for 30 min, incubated with the first primary antibodies (1:100) overnight at 4°C, rewarmed 30 min, and rinsed 3 times with PBS. Then the corresponding secondary antibodies (1:100) were added and incubated at room temperature for 1 h, rinsed 3 times with PBS. Incubate slides with CY3-TSA solution (diluted with TBST appropriately) for 10 min in dark condition. After that, wash slides with TBST three times, 5 min each time. Then the antigen repair procedure was repeated, and a second primary antibody and secondary antibody were successively added after three times of rinsing with PBS. DAPI incubation was performed in the dark for 5–10 min, following three cycles of washing with PBS, 1 min each cycle. The sections were sealed and placed under a microscope for photographing (34).

### 2.10 Western blot

Cells and tissues were lysed in radioimmunoprecipitation assay (RIPA) buffer (Thermo Fisher Scientific, 89900, USA) and boiled after detecting the concentration. Equal amounts of proteins were separated by 10% SDS-PAGE gels and transferred onto PVDF membranes (Bio-Rad, 1620177, USA). After blocking with 5% skimmed milk, membranes were incubated with anti-β-actin antibody (1:2000), anti-EV-A71 antibody (GTX132338, 1:2000), anti-ANXA2 antibody (GTX101902, 1:1000), anti-SCARB2(GTX53371, LIMP II) antibody (1:1000), anti-JAM-A antibody (GTX01112, 1:1000), anti-CLDN5 antibody (GTX00796, 1:1000), anti-OCLN antibody (GTX85016), anti-CLDN1 antibody (GTX134842) and anti-MMP-9 antibody (GTX100458, 1:1000) at 4°C overnight. After washing, the membranes were incubated with secondary HRP-conjugated antibodies for 1 h at RT. Then, the membranes were visualized with ECL Plus enhanced chemiluminescence. WB detection was carried out with a ChemiDoc MP imaging system (Bio-Rad, 12003154, USA). The relative protein expression was quantified using ImageJ software (Ver 1.51) (27).

### 2.11 Receptors (SCARB2 and ANXA2) blocking experiment

MVECs grown in 12-well plates were preincubated with anti-SCARB2 (GTX53371, 1:1000), anti-ANXA2 antibodies (GTX101902, 1:1000), anti-SCARB2 (1:1000) + anti-ANXA2 (1:1000), and anti-GAPDH (GTX100118, 1:1000) for 3 h at 37°C prior to infection. Then, the cells were infected with EV-A71 at an MOI of 0.1 for 2 h and washed with PBS once. Then, the cells were replenished with antibody-added medium and incubated for 24 h. The GAPDH blocking group was a negative control and the unblocked group was a blank control. The viral load was detected by RT-qPCR. The EV-A71 protein was detected by WB. The relative viral protein expression was quantified using ImageJ software (Ver 1.51) (35).

### 2.12 Statistical analysis

GraphPad Prism software version 8 was used for statistical analysis. Student’s t test, one-way ANOVA, or two-way ANOVA were used as appropriate. The quantified data are presented as the mean ± SD. A P-value of <0.05 was considered statistically significant (* P < 0.05, **P < 0.01, *** P < 0.001).

## 3 Results

In this study, we first established a model of tree shrews infected with EV-A71 *in vivo*. Tree shrews infected with EV-A71 showed some typical symptoms of HFMD. Importantly, EV-A71 invaded the central nervous system of tree shrews and increased the permeability of blood-brain barrier. Further studies found that EV-A71 could interact with SCARB2 and ANXA2 to enter the CNS and down-regulate CLDN5 and JAM-A. Then we proved that EV-A71 could directly infect the MVECs and AS in the brain of tree shrews. Receptors SCARB2 and ANXA2 are involved in the infection. EV-A71 infection down-regulated the expression of tight junction proteins, which may affect the permeability of blood-brain barrier.

### 3.1 EV-A71 infection induced virus-associated clinical characteristics in tree shrew models

According to our previous work (19), a nonlethal infectious dose of EV-A71 was used in this study. Some vesicular lesions manifested on the feet of infected tree shrew from 5 dpi to 14 dpi (**Figure 1B**). Following EV-A71 inoculation, the body temperature of tree shrews exhibited a marked increase from 5 dpi to 13 dpi (**Figure 1C**). There was a significant difference in the body temperature between the infection group and the control group (two-way repeated-measures ANOVA, P=0.043<0.05), indicating that EV-A71 infection can cause fever in tree shrews. Along with the increase in body temperature, the activity of the animals in the infection group was also observed to be significantly reduced. Since 3-month-old tree shrews were in their growth and development period, the body weight of the two groups increased during a three-week observation period (**Figure 1D**). But the weight gain of the infection group was significantly slower than that of the control group (two-way repeated-measures ANOVA, P<0.05).

The viral load in blood was detected every other day. It was very low at 1 dpi, but continued to increase in the first week. The viral load in blood reached a peak at 7 dpi, with a highest value of 1.31×10^5^ copies/mL (**Figure 1E**). The viral load was detectable in the blood of all tree shrews after EV-A71 inoculation, albeit at different levels. The viral load maintained a high level in the second week and then began to decrease, which was similar to the change trend of body temperature.

EV-A71 RNA was detected in most of the tissues taken at 7 dpi, including heart, liver, spleen, lung, kidney, muscles, sciatic nerve, brain, and spinal cord, while it is very little in muscles and sciatic nerves. At 20 dpi, it was mainly detected only in the tissues of the spleen, liver, lung, brain, and spinal cord (**Table 2**). Reduced viral loads in the brainstem from 7 to 20 dpi demonstrate progressive viral clearance, though clearance rates vary individually.

**Table 2:** Viral load in tissue samples from EV-A71-infected tree shrews.

Viral load (log_10_ copies/mg)	7dpi				20dpi			
	E1^*^	E4	E7	E12	E2	E5	E8	E10
Cerebrum	2.7^♯^	3.5	3.9	2.7	2.3	1.5	3.2	1.3
Cerebellum	2.7	3.4	2.7	2.3	1.0	3.0	2.8	—
Brain stem	1.2	3.5	3.5	1.3	2.7	3.0	1.8	—
Thoracic spinal cord	2.9	2.9	1.9	—	1.1	1.0	2.0	—
Lumbar spinal cord	2.9	2.1	1.7	—	—	0.8	1.9	—
Sciatic nerve	—	—	—	1.1	0.7	—	—	—
Upper-limb muscles	1.4	—	1.6	1.7	—	—	—	—
Lower-limb muscles	1.0	—	1.7	1.2	—	—	—	0.7
Heart	3.3	2.0	3.3	1.3	—	—	0.8	—
Lung	2.9	3.3	3.9	3.1	1.8	2.9	1.9	1.1
Liver	2.1	2.1	2.1	—	1.3	—	2.0	—
Spleen	1.9	2.7	2.5	1.3	1.2	2.1	1.7	—
Kidney	2.7	2.7	2.8	1.5	—	—	—	—
Small intestine	2.6	2.4	1.9	1.4	—	—	0.8	0.7
Large intestine	2.8	0.9	1.1	1.0	0.7	—	0.7	—
		0	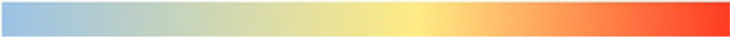				4	

*Animal code. # The copy number of viral genomic RNA is expressed as log_10_/mg. −Means undetectable.

The color intensity is proportional to viral load from low (light blue) to high (red).

### 3.2 EV-A71 infection induced pathological changes in various tissues of tree shrews, including the CNS

**Figure 2:**
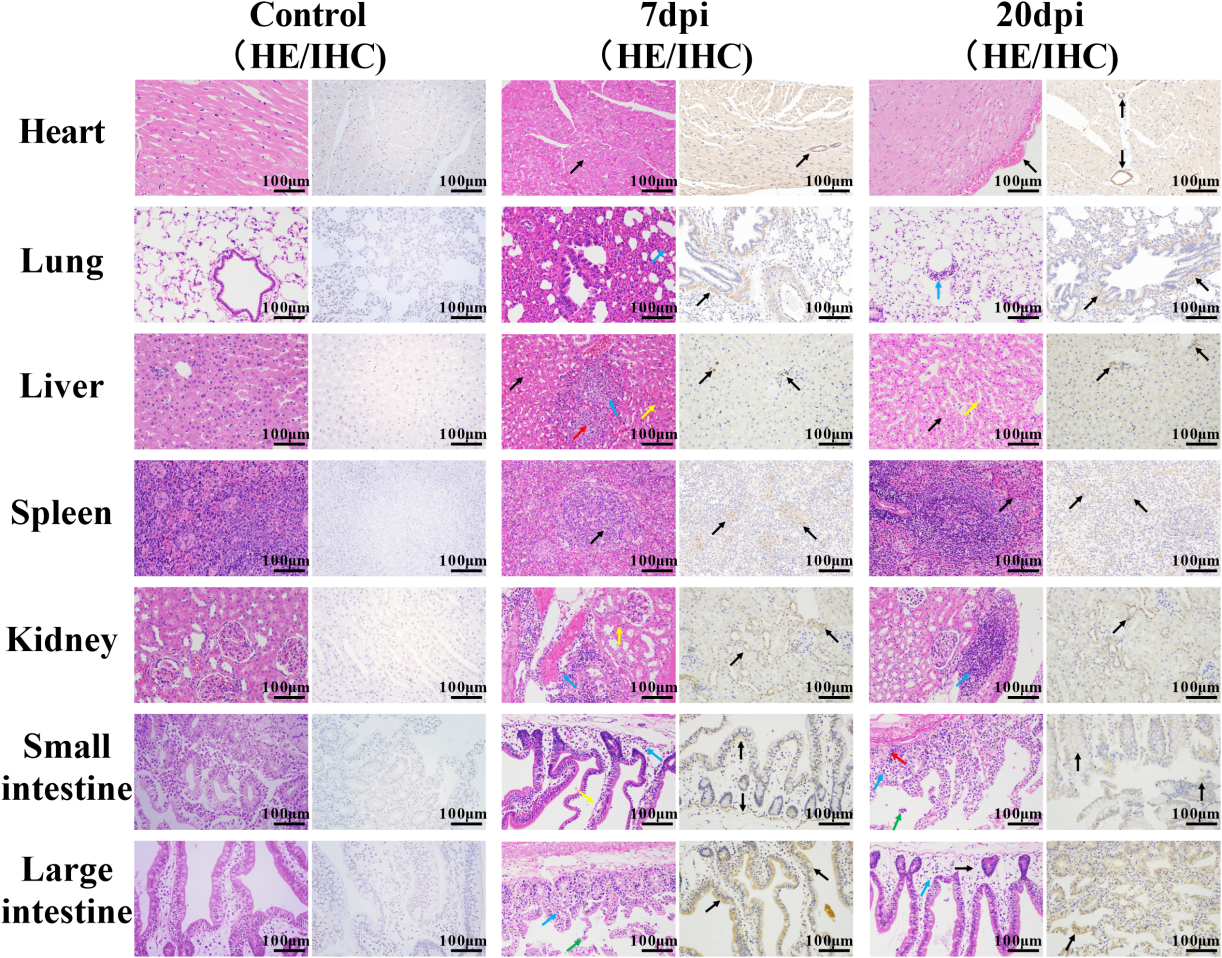
Histopathological characterization of non-neural tissues. Scale bar=100 μm. 7 dpi: Heart, cell degeneration (black arrows); lung, alveolar walls thickened, alveolar cavity narrowed, and inflammatory cell infiltration (blue arrow); liver, fatty degeneration, cytoplasmic vacuolation (black arrows), hepatic sinusoids slightly congested and dilated (yellow arrow), necrotic foci (red arrow), and granulocyte infiltration (blue arrow); spleen, apoptotic bodies (black arrows), granulocyte infiltration (blue arrow); kidney, multiple congestion (yellow arrow), inflammatory cell infiltration (blue arrow); small intestine and large intestine, enterocytes separated from the lamina propria (yellow arrow), shed cell debris (green arrow), inflammatory cell infiltration (blue arrow). 20 dpi: Heart/spleen, tissue congestion (black arrows); lung/kidney, inflammatory cell infiltration (blue arrows); liver, hepatic sinusoids congested and dilated (yellow arrow); small intestines and large intestines, cell debris (green arrow), severe edema (black arrow), inflammatory cell infiltration (blue arrow) and cytoplasmic vacuolation (red arrow). Immunohistochemical staining showed the distribution of EV-A71 protein (black arrows) in different tissues.

**Figure 3:**
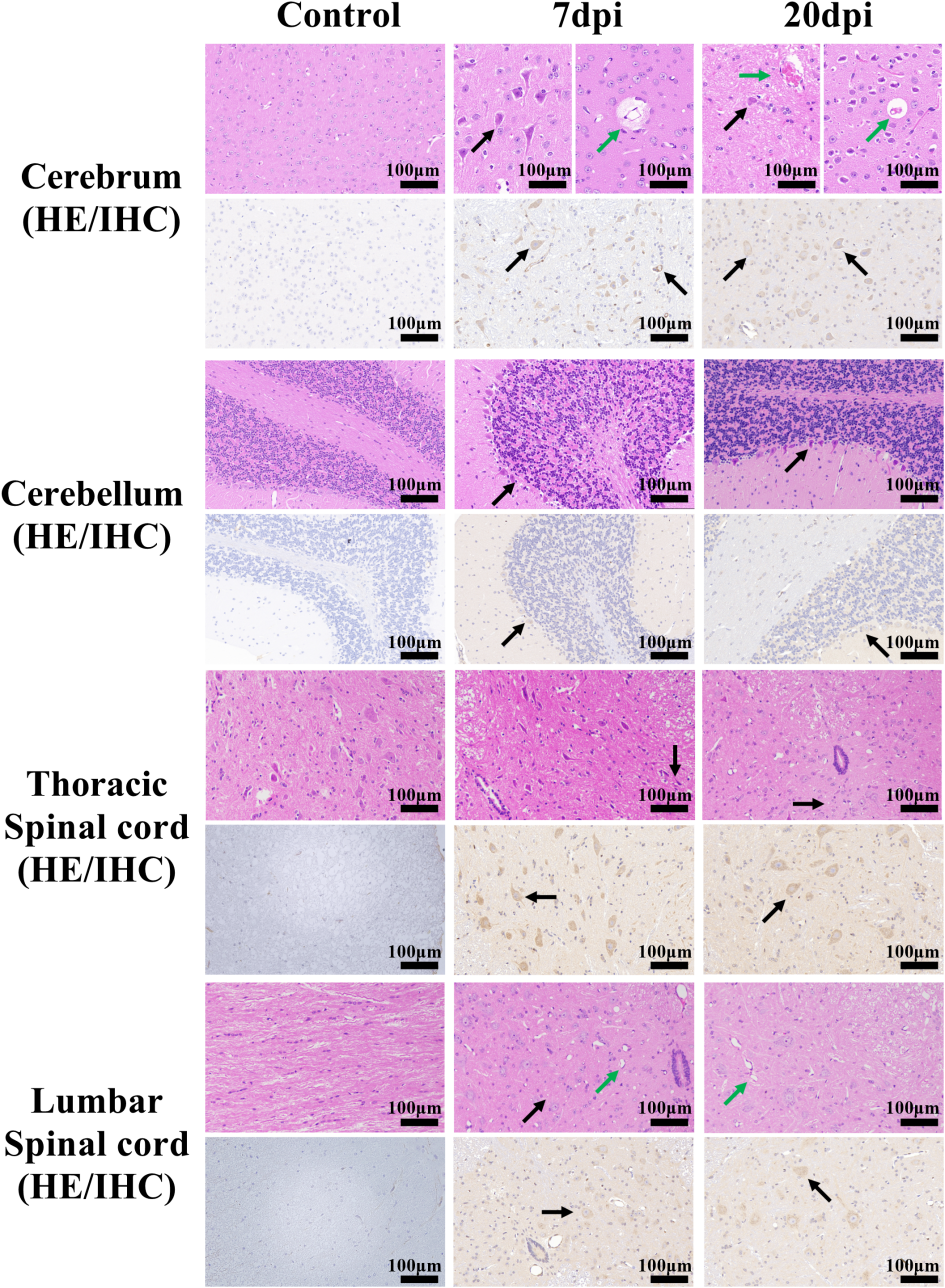
Histopathological and immunohistochemical examination of the brain and spinal cord tissues. Scale bar=100 μm. 7 dpi: Cerebrum (mainly cerebral cortex and cerebral medulla, the same below), perivascular edema (green arrow); Cerebrum, cerebellum and thoracic spinal cord, neurons pyknotic and deeply stained, with irregular shapes (black arrow); lumbar spinal cord, vasodilatation (green arrow) and cellular vacuoles (black arrow). 20 dpi: Cerebrum, Vasodilation and Vascular endothelial cell necrosis (green arrow); Cerebrum and cerebellum, neurons pyknotic and deeply stained, with irregular shapes (black arrow); thoracic spinal cord and lumbar spinal cord, cellular vacuoles (black arrow) and vasodilatation (green arrow). Immunohistochemical staining showed the distribution of EV-A71 protein (black arrows) in different tissues.

Histopathological examination revealed that at 7 dpi and 20 dpi, all major organs showed some pathological changes (**Figures 2**, **3**), compared to the control group. All histopathological examinations are summarized in **Table 3**. At 7 dpi, some cardiomyocytes of the heart (3/4) showed cell degeneration, cell swelling, cytoplasmic looseness and light staining. A large number of alveolar walls were severely thickened (2/4), and the alveolar cavity was narrowed, accompanied by a moderate amount of inflammatory cell infiltration. A large number of hepatocytes (3/4) had fatty degeneration, and small round vacuoles appeared in the cytoplasm. Hepatic sinusoids were slightly congested and dilated. There were multiple necrotic foci, where the nucleus shrank, fragmented or dissolved and disappeared, cytoplasmic eosinophils also increased, and there was a small amount of granulocyte infiltration around it. Apoptotic bodies were common in the white pulp of the spleens (4/4); the red pulp was slightly congested with a small amount of granulocyte infiltration. Multiple congestion was seen in the kidney (4/4), and inflammatory cell infiltration was common in the renal pelvis. In both the small and large intestine, a large number of intestinal villi and epithelial cells sloughed off, and notable amounts of shed cell debris were seen in the intestinal lumen; the lamina propria intestinal glands were arranged irregularly, accompanied by a small amount of inflammatory cell infiltration. In the cerebrum, cerebellum and spinal cord, a large number of neurons (2/4) were pyknotic and deeply stained, with irregular shapes. Vasodilation, perivascular edema and endothelial cell necrosis can be seen in the brain and spinal cord. No significant pathological alterations were observed in the muscle tissue or sciatic nerves.

**Table 3:** Summary of histopathological examination of tissues from 8 EV-A71-infected tree shrews.

Tissues	7dpi						20dpi					
	HE			IHC:H-score			HE			IHC:H-score		
	^−^	^+^	^++^	EV-A71	ANXA2	SCARB2	^−^	^+^	^++^	EV-A71	ANXA2	SCARB2
Brain		2/4^*^	2/4	130.63	76.22	37.81	2/4	1/4	1/4	101.51	63.52	34.88
Cerebellum		3/4	1/4	124.57	86.24	48.86	2/4	2/4		71.85	55.64	27.08
Thoracic spinal cord		2/4	2/4	37.52	14.44	51.67	4/4			29.94	12.23	26.61
Lumbar spinal cord	1/4	3/4		21.30	10.43	30.10	4/4			4.59	14.35	16.56
Sciatic nerve	4/4			14.82	15.71	31.54	4/4			6.69	12.03	14.05
Upper-limb muscles	4/4			//	//	//	4/4			//	//	//
Lower-limb muscles	4/4			//	//	//	4/4			//	//	//
Heart	1/4	3/4		56.07	//	//	3/4	1/4		14.82	//	//
Lung		2/4	2/4	61.77	//	//	3/4	1/4		37.52	//	//
Liver		3/4	1/4	100.12	//	//	2/4	2/4		15.71	//	//
Spleen		4/4		24.05	//	//	2/4	2/4		31.54	//	//
Kidney		4/4		98.79	//	//	3/4	1/4		10.43	//	//
Small intestine		1/4	3/4	100.60	//	//	2/4	2/4		51.67	//	//
Large intestine		1/4	3/4	91.78	//	//	2/4	2/4		30.10	//	//

*The ratio of animals with pathological changes over all dissected animals.

- No abnormality, + Mild abnormality, ++ Moderate abnormality.

// Indicates no data.

The H-SCORE is 0–300, with a higher score representing stronger positive staining.

At 20 dpi, the overall pathological changes were mild, mainly including tissue congestion (heart/spleen) and inflammatory cell infiltration (lung/kidney). In the liver, small round vacuoles appeared in the cytoplasm, and hepatic sinusoids were slightly congested and dilated. In the small and large intestines, cell debris, severe edema and inflammatory cell infiltration were common, and some cytoplasm became vacuole. Small numbers of neurons were pyknotic and deeply stained, with irregular shapes in the cerebrum and cerebellum.

Immunohistochemical examination also revealed the distribution of EV-A71 antigen in different tissues, which was basically consistent with the pathological changes.

### 3.3 Infection of EV-A71 increased the permeability of BBB in tree shrew models

**Figure 4:**
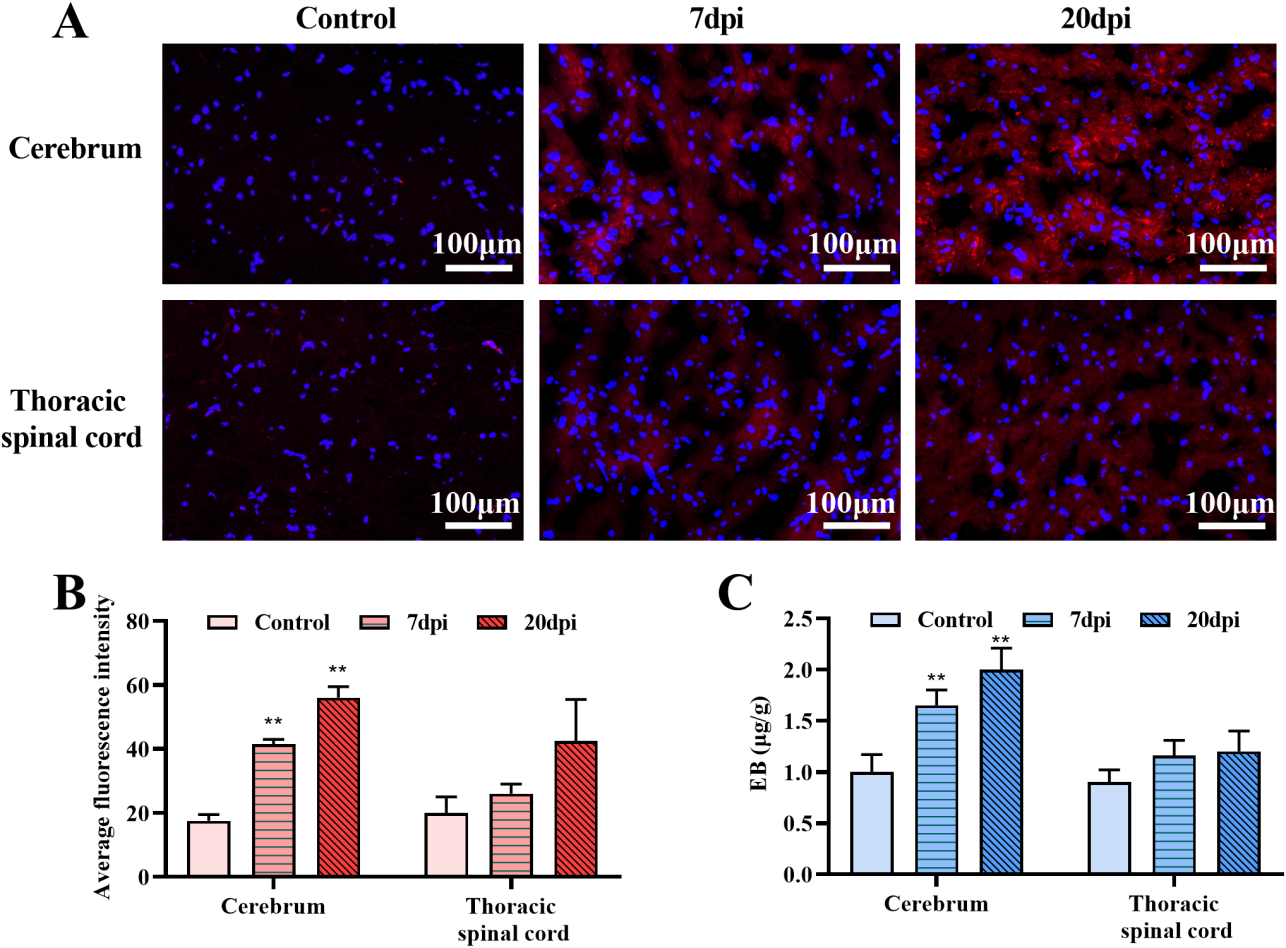
Evans blue (EB) penetration test in the cerebrum and thoracic spinal cord. **(A)** The fluorescence distribution of EB in control group and EV-A71group (7dpi, 20dpi). **(B)** Average fluorescence intensity calculated by ImageJ. **(C)** EB content in tissues. **P < 0.01 (One-way ANOVA, number of replicates = 3, error bars represent SD).

EB is a commonly used BBB permeability indicator that shows red fluorescence under a fluorescence microscope. The microscopic examination results for frozen cerebrum and thoracic spinal cord sections are shown in **Figure 4A** and **Supplementary Figure 2**. Compared with the control group, the fluorescence range in the EV-A71 infected group was significantly expanded. Then, five photos were chosen for each sample, covering different areas from the edge to the center, and ImageJ was used to calculate the average fluorescence intensity. Semiquantitative data (**Figure 4B**) showed that the fluorescence intensity in the cerebrum and thoracic spinal cord increased after infection, and notable changes were detected in the cerebrum at both 7 and 20 dpi (P<0.01). This result was supported by EB content determination (**Figure 4C**). The amount of EB in the brain of the infected group was about 1.5 times (7 dpi) and twice (20 dpi) that of the control group, which was also a significant difference (P<0.01). The above results indicated that EV-A71 infection can increase the permeability of the BBB.

### 3.4 EV-A71 infection in the brain was associated with receptors SCARB2 and ANXA2 and down-regulated the tight junction proteins CLDN5 and JAM-A

In order to further explore how EV-A71 enters the CNS and affects BBB, we conducted studies of receptors and tight junction proteins. SCARB2, ANXA2 and P-Selectin Glycoprotein Ligand 1(PSGL-1)are known functional receptors for EV-A71, and PSGL-1 is mainly expressed on leukocytes (36), so we only studied SCARB2 and ANXA2 here. CLDN1, CLDN5, OCLN and JAM-A were the mainly concerned tight junction proteins.

**Figure 5:**
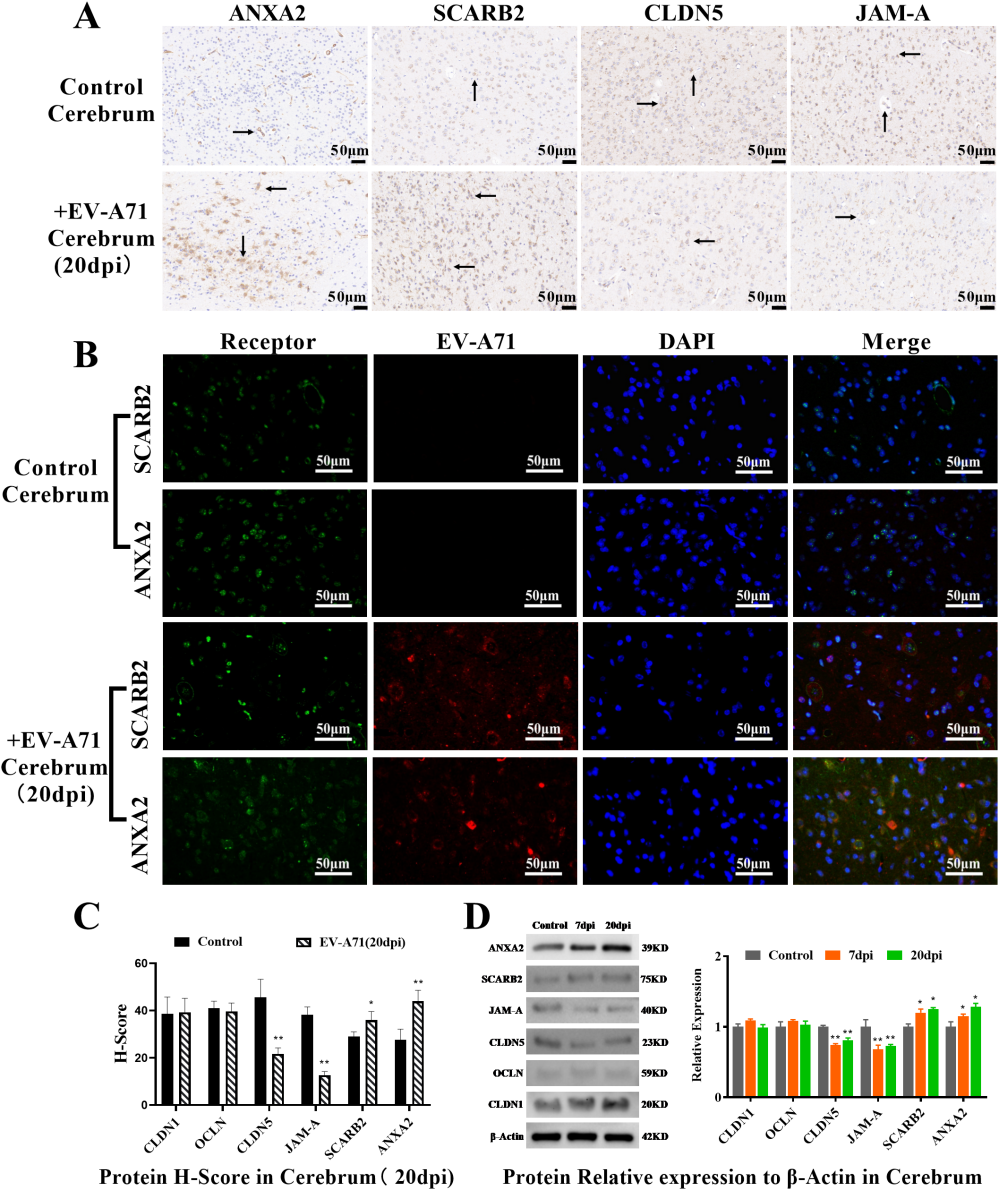
Observation and quantification of the receptors and tight junction proteins in cerebrum. **(A)** Immunohistochemical examination, scale bar=50 μm; **(B)** Double immunofluorescence, scale bar=50 μm. **(C)** Protein H-Score (T-test, * P < 0.05, **P < 0.01, n=3, error bars represent SD); **(D)** Quantification of the receptors and tight junction proteins by WB (One-way ANOVA, number of replicates = 3, error bars represent SD, * P < 0.05, **P < 0.01).

Immunohistochemical observation and scoring of the brain tissue (**Figure 5A**, **Supplementary Figure 3**) showed that the expression of receptors increased and the expression of CLDN5 and JAM-A decreased in the infected group, which was significantly different from that in the control group (P<0.01). Double immunofluorescence showed that the distribution of receptors ANXA2 and SCARB2 were related to that of EV-A71 in the brain (**Figure 5B**), and this is consistent with the distribution of EV71 receptor SCARB2 in human tissues (37). H-Score and WB results (**Figures 5C, D**, **Supplementary Figure 4**) also confirmed that CLDN5 and JAM-A were significantly down-regulated (P<0.01) and receptors were significantly up-regulated in the infected group (P<0.05).

### 3.5 EV-A71 infection caused cytopathic effect in the primary tree shrew brain MVECs and AS *in vitro*

MVECs and tight junctions provide the basic structure of the blood-brain barrier, and AS play an important role in maintaining the normal structure and function of the blood-brain barrier (38, 39). MVECs and AS can be used to investigate the possibility of EV-A71 passing through the blood-brain barrier *in vitro*.

**Figure 6:**
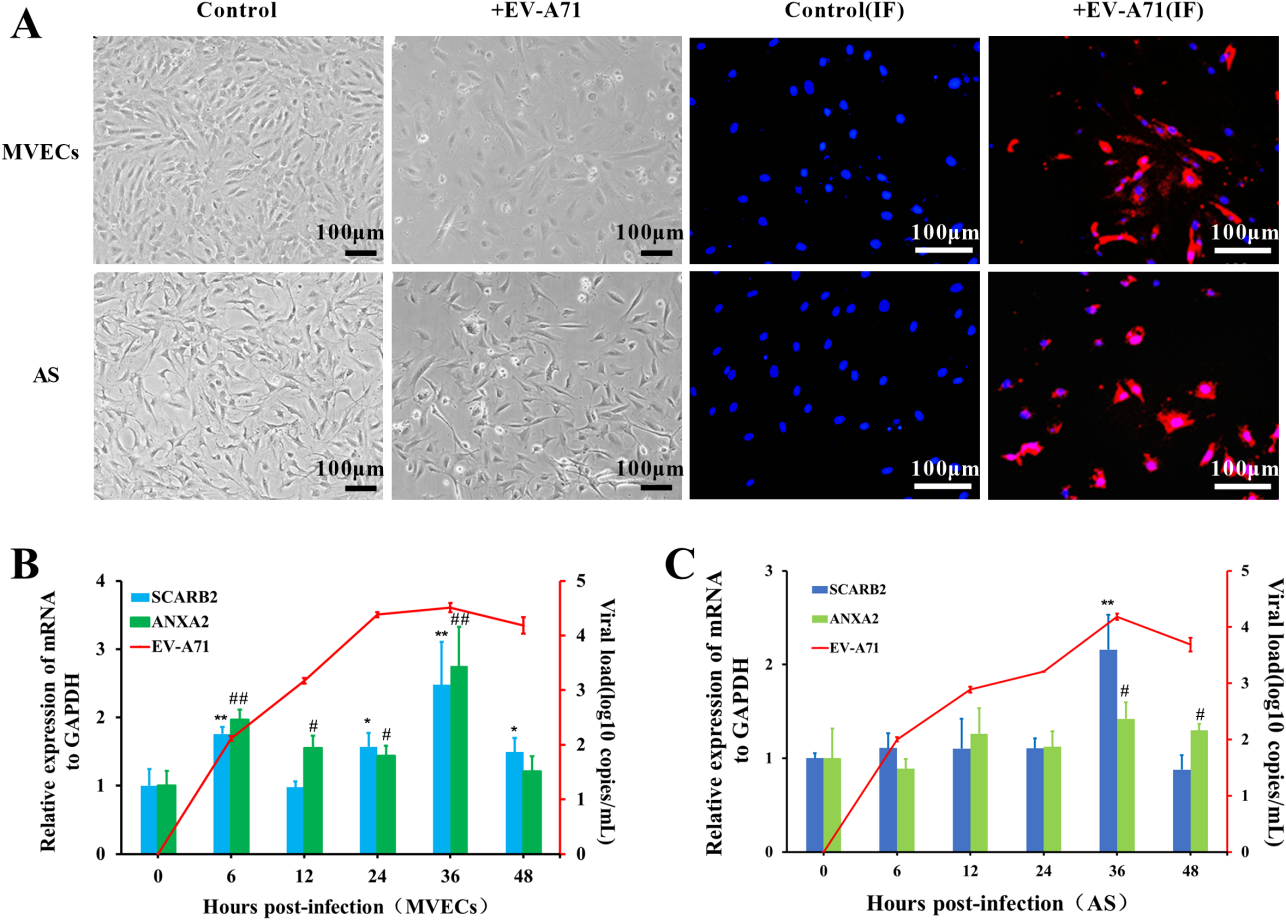
Cytopathic effect (CPE) of tree shrew brain MVECs and AS infected with EV-A71. **(A)** CPE observation, EV-A71 is indicated by red fluorescence, and the nucleus is indicated by blue fluorescence (MOI=0.5, 24 hpi, scale bar=100 μm). **(B, C)** Viral load of EV-A71 and Relative mRNA expression of receptors in MVECs and AS (One-way ANOVA, number of replicates = 3, error bars represent SD, */# P < 0.05, **/##P < 0.01).

As shown in **Figure 6A**, 24h post infection, many cells become rounded and floating, and the cell space becomes larger, compared to the control. The CPE was also supported by immunofluorescence experiments. Red fluorescence distribution was observed in both MVECs and AS, indicating that EV-A71 successfully infected the two kinds of cells.

To further analyze the infection characteristics of EV-A71 in MVECs and AS, cell RNA was extracted at 0, 6, 12, 24, 36 and 48 hpi, and the viral load was detected by TaqMan RT-qPCR. Using GAPDH as the internal control, the relative mRNA expression of the receptors was detected by SYBR Green RT-qPCR. The virus replicated rapidly in the first 24 h and then slowed down, reaching a peak at approximately 36 hpi (**Figures 6B, C**). In MVECs, the mRNA of both SCARB2 and ANXA2 was significantly up-regulated at 6hpi, 24hpi and 36hpi (P<0.05), but SCARB2 expression was stable at 12 hpi. In AS, significant up-regulation of receptors was mainly at 36 hpi. Preliminary evidence indicated that the two receptors were involved in EV-A71 infection.

### 3.6 EV-A71 infected tree shrew MVECs and AS *in vitro* through interacting with its receptors SCARB2 and ANXA2

**Figure 7:**
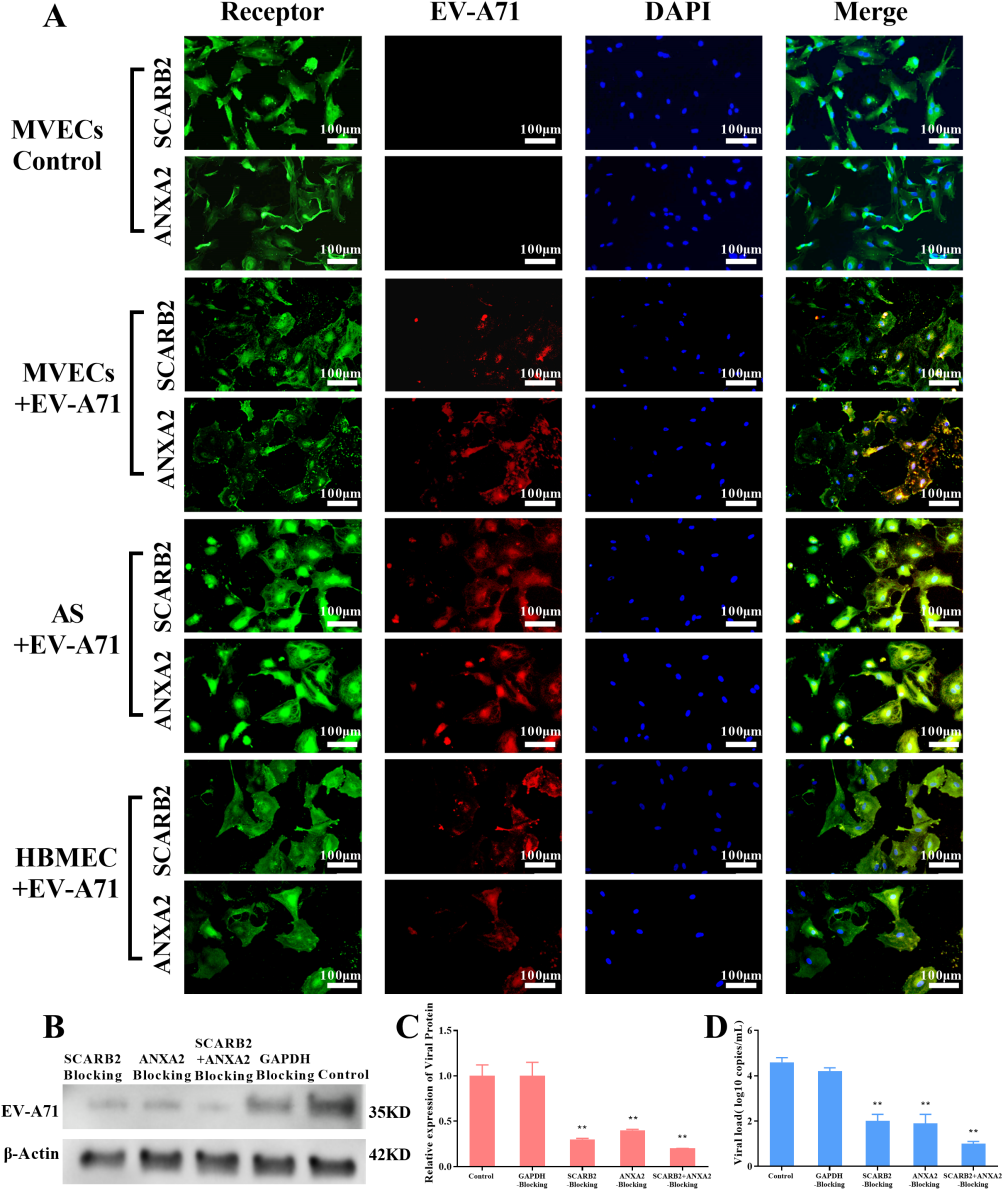
Immunofluorescence observation and blocking experiment of EV-A71 receptors (MOI=0.1, 24 hpi, scale bar=100 μm). **(A)** Immunofluorescence observation, green fluorescence represents the receptors SCARB2 and ANXA2, and red fluorescence represents EV-A71, HBMEC+EV-A71 was the positive control, MVECs was the blank Control. **(B)** WB of EV-A71 protein in different blocking groups of MVECs, GAPDH blocking group was a negative control and the unblocked group was a blank control. **(C-D)** Relative viral protein expression, using ImageJ and viral load by RT-qPCR (One-way ANOVA, number of replicates = 3, error bars represent SD, **P < 0.01).

To further determine the role of the two receptors, a double immunofluorescence observation was first performed. As shown in **Figure 7**, green fluorescence represented the receptor, and red fluorescence represented EV-A71. It was clearly observed that the virus and the receptor overlap in many parts of the cell. The results of tree shrew brain MVECs and AS were consistent with the positive control HBMECs.

Then, to confirm the role SCARB2 and ANXA2 play in EV-A71 infection, a blocking experiment was carried out in tree shrew brain MVECs. The cells were pretreated with antibodies against SCARB2 and ANXA2, individually and in combination for 3 h, and then were infected with EV-A71. 24h after infection, the viral load and viral protein expression in the blocking groups were significantly reduced (P<0.01), compared with the GAPDH blocking group and control group (**Figures 7B-D**). The results indicated that SCARB2 and ANXA2 played important roles in EV-A71 infection.

### 3.7 EV-A71 infection down-regulates the expression of tight junction proteins and up-regulates the expression of immune cytokines in tree shrew brain MVECs and AS

To further analyze the impact of EV-A71 in MVECs and AS, we determined the expression levels of tight junction proteins and immune cytokines by RT-qPCR and WB.

**Figure 8:**
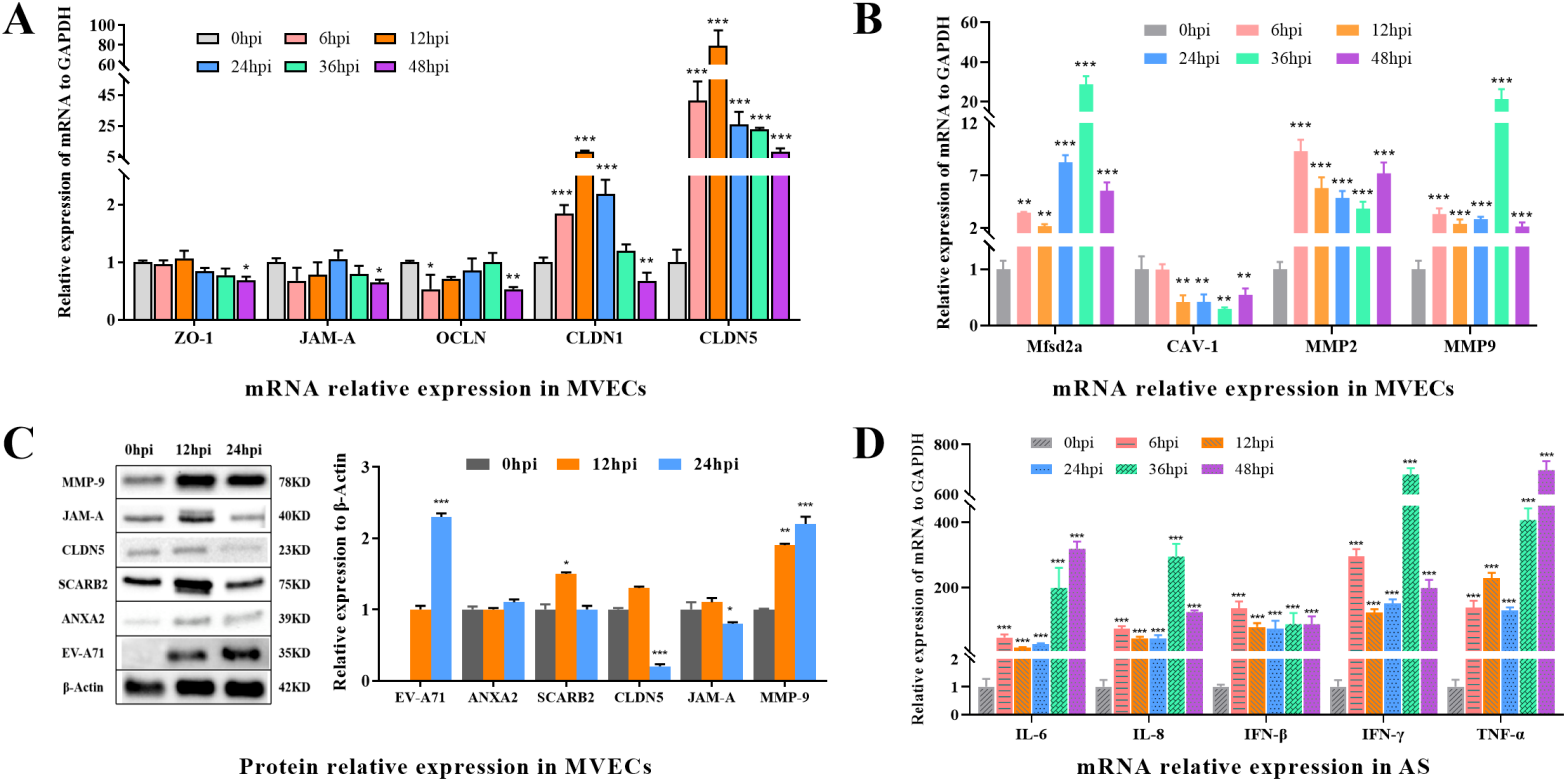
The expression of BBB-related proteins, EV-A71, receptors, and cytokines in MVECs and AS. **(A)** Relative mRNA expression of tight junction proteins, including CLDN1, CLDN5, OCLN, ZO-1 and JAM-A in MVECs. **(B)** Relative mRNA expression of Mfsd2a, CAV-1, MMP2 and MMP9 in MVECs. **(C)** WB and relative protein expression in MVECs using ImageJ. **(D)** Relative mRNA expression of cytokines, including IL-6, IL-8, IFN-β, IFN-γ and TNF-α, in AS. (One-way ANOVA, number of replicates = 3, error bars represent SD, *P < 0.05, **P < 0.01, ***P < 0.001.).

As illustrated in **Figure 8**, the mRNA expression levels of ZO-1, JAM-A, OCLN, and CLDN1 were significantly down-regulated at 48hpi (P<0.05). Both CLDN1 and CLDN5 demonstrated a pronounced upward trend from 6 to 12 hpi, reaching several times to 50 times that of the control (0hpi), and then went down to the end. WB results showed that JAM-A and CLDN were significantly down-regulated (P<0.05) at 24 hpi, consistent with expression changes in brain tissue. Despite some differences in mRNA and protein expression levels, it was speculated that EV-A71 infection down-regulates tight junction proteins in MVECs.

Major facilitator superfamily domain containing 2a (Mfsd2a) regulates vesicle transport, Caveolin 1 (CAV-1) is involved in vesicle transport, and the activation of matrix metalloproteinases (MMPs) also affects the normal function of the BBB. After infection, Mfsd2a, MMP2 and MMP9 showed significantly up-regulation (P<0.05), while CAV-1 expression was significantly down-regulated (P<0.05), indicating that vesicle transport efficiency or barrier permeability was affected.

In terms of mRNA expression, the cytokines Interleukin 6 (IL-6), Interleukin 8 (IL-8), Interferon Beta (IFN-β), Interferon Gamma (IFN-γ), and Tumor Necrosis Factor Alpha (TNF-α) were significantly upregulated (P < 0.001) to varying degrees following infection. This preliminary observation suggests that EV-A71 infection may enhance the cellular immune activity of AS. This finding aligns with previous research on EV-A71 infection in rhesus monkeys (40), which demonstrated that EV-A71 preferentially targets astrocytes in the central nervous system, resulting in the upregulation of IL-6, IL-8, and IFN-γ. To further elucidate these effects, future studies should employ more precise detection methods, such as ELISA.

## 4 Discussion

According to literature reports, tree shrews are an emerging viral infection model that has been widely used in research on hepatitis viruses, respiratory viruses, arboviruses, and other viruses (41). Our study establishes the first comprehensive *in vivo* and *in vitro* tree shrew model for investigating EV-A71 neuropathogenesis. Notably, three-month-old infected tree shrews developed hallmark clinical features of HFMD—including vesicular lesions, febrile responses, and growth retardation—closely mirroring pediatric manifestations (42). Furthermore, the virus demonstrated replication in the bloodstream and major organs, including the brain and spinal cord, aligning with the findings from EV-A71 infection in rhesus monkeys (43).

According to reports in the literature, clinical pathological assessments of patients infected with EV-A71 have demonstrated pulmonary edema, lung tissue damage, and inflammatory cell accumulation (44–46), in addition to varying degrees of myocarditis (47). Histopathological analyses of EV-A71-infected tree shrews have further corroborated the clinical relevance of this model. Our investigation demonstrated that following infection, the lung tissue exhibited congestion and enlargement of the alveolar septum. Additionally, we identified various phenomena, such as the presence of cell vacuoles and lymphocyte infiltration, in certain non-neural tissues. Within the central nervous system (CNS), a substantial quantity of pyknotic and intensely stained neurons, as well as vacuolar degeneration, were distinctly observed. The results of this study exhibit similarities to the observed tissue distribution in rhesus monkeys (20), cynomolgus monkeys (48, 49), and mice (50) after EV-A71 infection. These congruences position the tree shrew as a uniquely predictive model for EV-A71 neurotropism.

Viruses like the Japanese encephalitis virus (JEV) compromise blood-brain barrier (BBB) integrity by disrupting tight junctions (TJs). JEV affects claudin-1 expression, leading to increased barrier permeability (51). In JEV-infected mice, BBB impairment is linked to reduced expression of TJ genes like CLDN5, OCLN, and ZO-1 (52). Similarly, EV-A71-infected tree shrews show TJ disruption, with elevated BBB permeability and decreased CLDN5 and JAM-A expression, as demonstrated by Evans blue assays. This disruption likely facilitates viral neuroinvasion (53), as supported by *in vitro* evidence of productive EV-A71 infection in primary brain MVECs and astrocytes—key cellular constituents of the BBB. Receptor co-localization studies identified SCARB2 and ANXA2 as putative mediators of viral entry, consistent with their established roles in human EV-A71 pathogenesis (36, 54). Importantly, our inoculation route (oral/nasal) recapitulates natural transmission pathways, avoiding the artifactual intramuscular delivery used in rodent models that exacerbates muscle tropism (12, 13). The observed viremia dominance over peripheral nerve/muscle replication strongly supports hematogenous CNS invasion, contrasting with murine axonal transport paradigms (12).

While our findings implicate BBB breakdown as a primary neuroinvasive mechanism, three critical limitations warrant consideration. First, our receptor analysis has limitations. EV-A71 receptors include SCARB2, PSGL-1, ANXA2, vimentin, nucleolin, and heparan sulfate proteoglycans (55). SCARB2 facilitates virus binding, internalization, and uncoating, and its monoclonal antibody JL2 can block binding and inhibit cell damage (14, 56). PSGL-1, mainly in immune cells, aids virus entry but not shell shedding; its antibody can block binding to reduce cell death (36). ANXA2, found in endothelial cells, acts as an attachment receptor for co-infection (57). CyclopA also aids virus entry (58). This study focuses on brain MVECs and astrocytes (non-immune cells), limiting receptors to SCARB2 and ANXA2, which are widely expressed, including in the brain. Additionally, due to the lack of specific antibodies, data for other receptors are unavailable. Second, although receptor blocking experiments attenuated viral replication, genetic validation through CRISPR knockout or overexpression remains essential to confirm SCARB2/ANXA2 functionality. Third, while transcriptomic data suggested EV-A71-mediated dysregulation of MMPs and Mfsd2a—potential modulators of BBB integrity—proteomic validation is required to establish causal relationships.

Viral stimulation induces a substantial release of inflammatory mediators, such as IFN-α, IL-8, TNF-α, and IL-6, which contribute to increased permeability of the BBB (59, 60). In murine models of JEV, elevated levels of IL-6 have been shown to alter TJ structures, thereby compromising BBB integrity (61). Consequently, the upregulation of inflammatory factors resulting from EV-A71 infection in tree shrew AS may indirectly affect BBB functionality, although further empirical validation is necessary. Furthermore, recent *in vitro* evidence proposing lymphocyte-facilitated neuroinvasion (62) highlights the need for integrated models incorporating immune components, a direction our future studies will pursue.

## 5 Conclusions

In summary, our study demonstrated that EV-A71 is capable of traversing the blood-brain barrier (BBB) to infect the brain and induce central nervous system (CNS) damage in tree shrew models. Mechanistically, EV-A71 was found to infect and exert cytopathic effects on the brain microvascular endothelial cells (MVECs) and astrocytes (AS) of tree shrews by binding to receptors such as SCARB2 and ANXA2. This interaction further compromised the integrity of the BBB, thereby increasing its permeability in the tree shrew brain. By elucidating these critical mechanisms underlying BBB disruption and neuronal injury in a relevant model, our findings establish a mechanistic basis for the development of therapeutic strategies aimed at preventing EV-A71 entry into the CNS and enhancing BBB protection.

## Data Availability

The original contributions presented in the study are included in the article/**Supplementary Material**. Further inquiries can be directed to the corresponding author/s.

## Supplementary material

Supplementary Figure 1 Standard curves for TaqMan RT-qPCR.

Supplementary Figure 2 The fluorescence distribution of EB in the cerebrum and thoracic spinal cord (High-definition images for **Figure 4A**).

Supplementary Figure 3 Immunohistochemical examination of the receptors and tight junction proteins in cerebrum (High-definition images for **Figure 5A**).

Supplementary Figure 4 Western blot images of the receptors and tight junction proteins in cerebrum (**Figure 5**) **(A)** ANXA2,39KD. **(B)** SCARB2,75KD. **(C)** JAM-A,40KD. **(D)** CLDN5,23KD. **(E)** OCLN,59KD. **(F)** CLDN1,20KD. **(G)** β-Actin, 42KD.
