# Supplementary figures and images for: EV-A71 invades the central nervous system and affects the blood-brain barrier in a tree shrew model
Source: Front Immunol. 2025 Jun 26;16:1583768. doi: 10.3389/fimmu.2025.1583768 (PMC12241082; doi:10.3389/fimmu.2025.1583768)

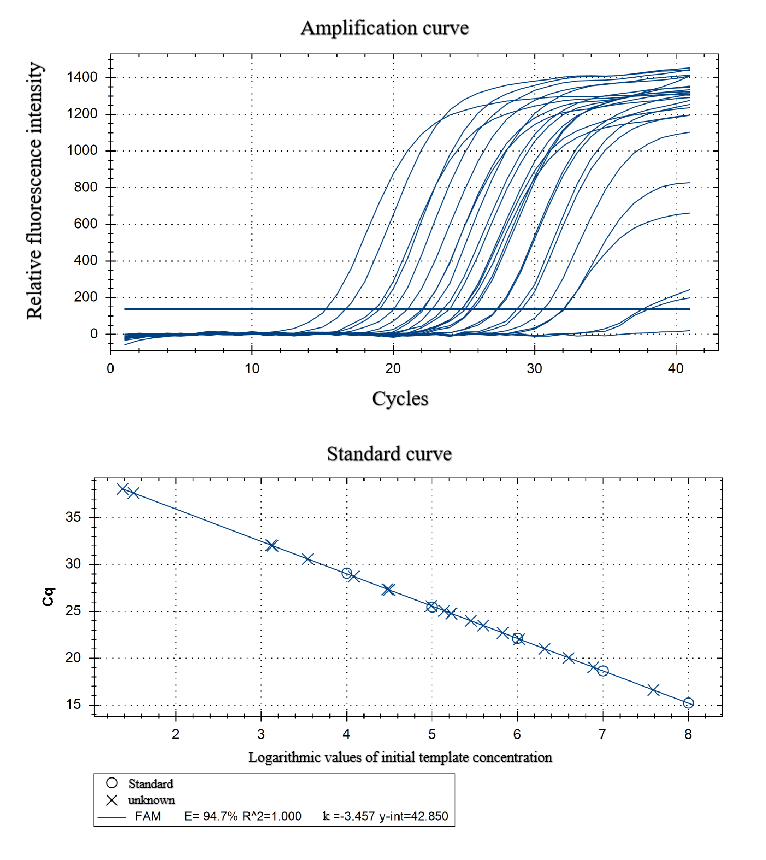

Supplement: Supplementary Figure 1 — Standard curves for TaqMan RT-qPCR. [file Image1.tif]

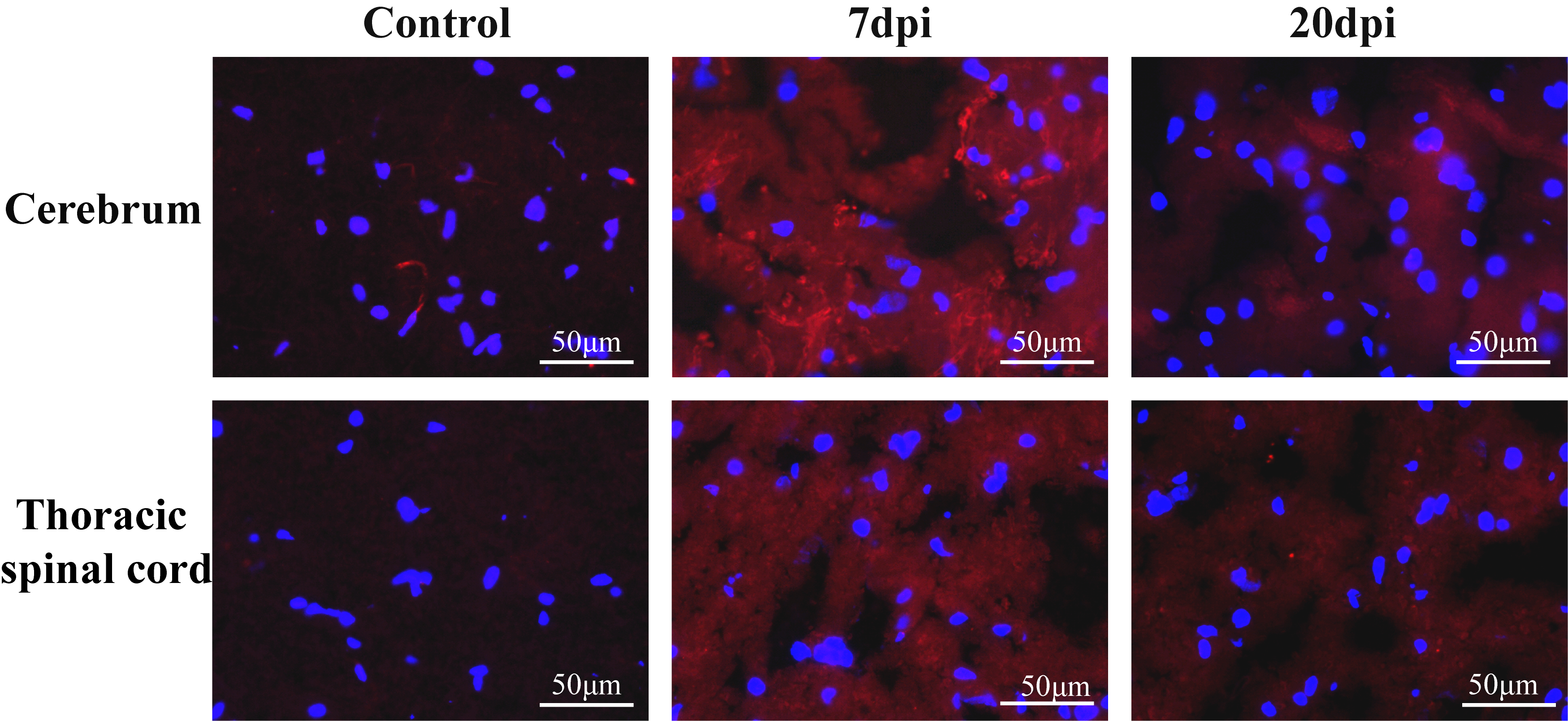

Supplement: Supplementary Figure 2 — The fluorescence distribution of EB in the cerebrum and thoracic spinal cord (High-definition images for Figure 4A ). [file Image2.tif]

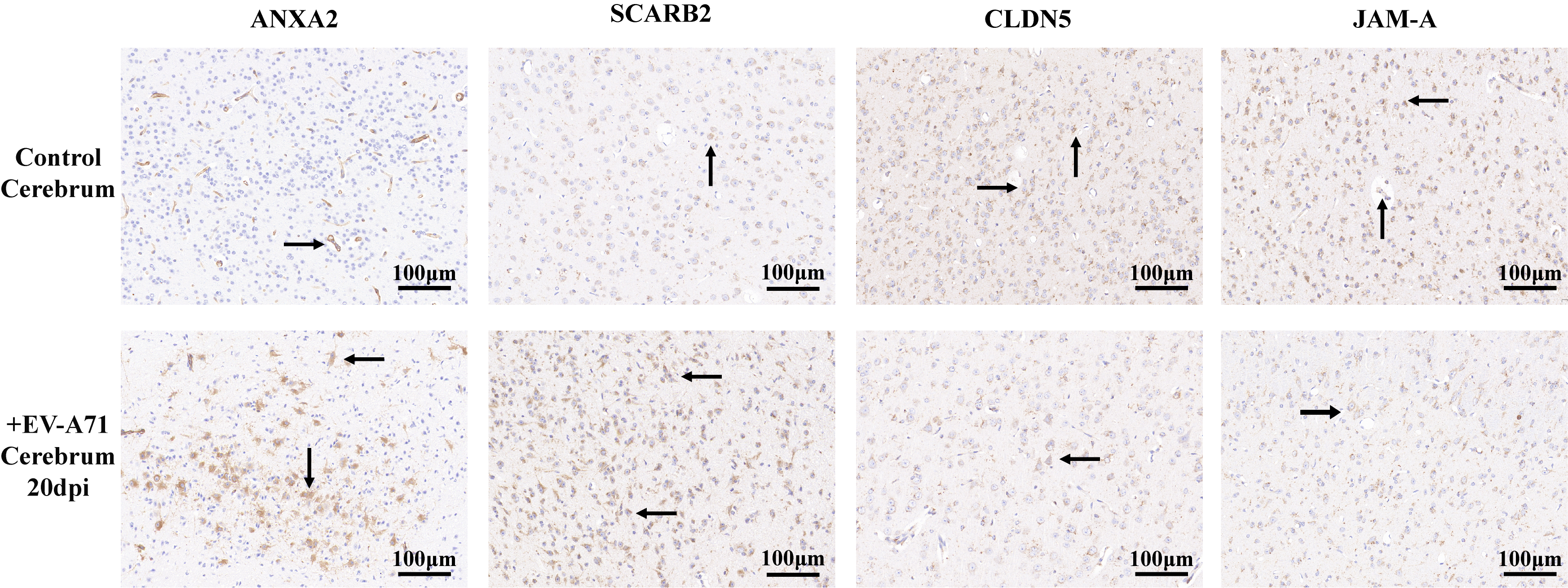

Supplement: Supplementary Figure 3 — Immunohistochemical examination of the receptors and tight junction proteins in cerebrum (High-definition images for Figure 5A ). [file Image3.tif]

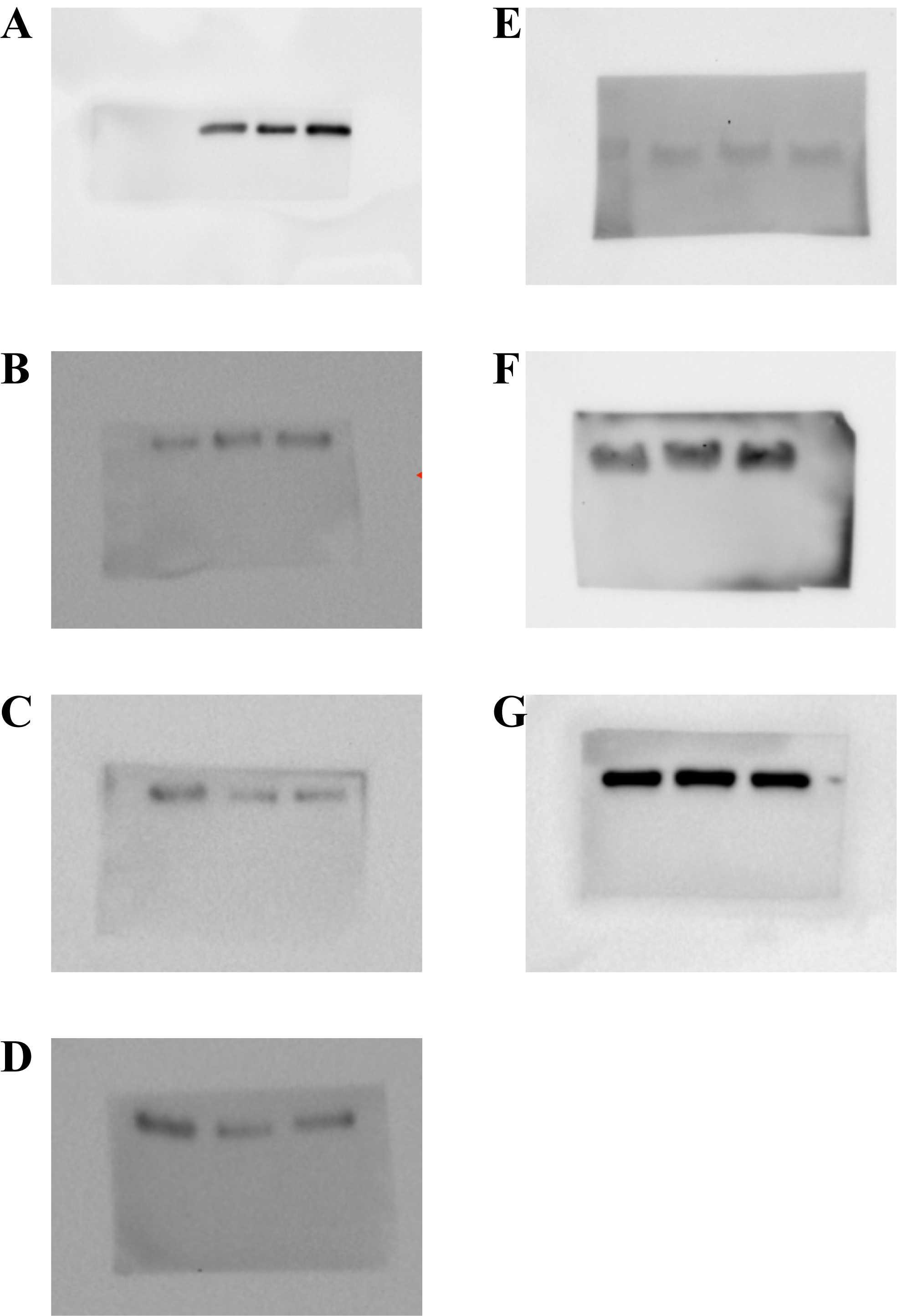

Supplement: Supplementary Figure 4 — Western blot images of the receptors and tight junction proteins in cerebrum ( Figure 5 ) (A) ANXA2,39KD. (B) SCARB2,75KD. (C) JAM-A,40KD. (D) CLDN5,23KD. (E) OCLN,59KD. (F) CLDN1,20KD. (G) β-Actin, 42KD. [file Image4.tif]
